# A unified approach to inferring chemical compounds with the desired aqueous solubility

**DOI:** 10.1186/s13321-025-00966-w

**Published:** 2025-03-26

**Authors:** Muniba Batool, Naveed Ahmed Azam, Jianshen Zhu, Kazuya Haraguchi, Liang Zhao, Tatsuya Akutsu

**Affiliations:** 1 Discrete Mathematics and Computational Intelligence Laboratory, Department of Mathematics, Quaid-i-Azam University, Islamabad, Pakistan; 2 Discrete Mathematics Laboratory, Department of Applied Mathematics and Physics, Graduate School of Informatics, Kyoto University, 606-8501 Kyoto, Japan; 3 Graduate School of Advanced Integrated Studies in Human Survivability (Shishu-Kan), Kyoto University, 606-8306 Kyoto, Japan; 4 Bioinformatics Center, Institute for Chemical Research, Kyoto University, 611-0011 Uji, Japan

**Keywords:** Molecular design, QSAR/QSPR, Machine learning, Integer programming, Graph-theoretic descriptors, Aqueous solubility

## Abstract

Aqueous solubility (AS) is a key physiochemical property that plays a crucial role in drug discovery and material design. We report a novel unified approach to predict and infer chemical compounds with the desired AS based on simple deterministic graph-theoretic descriptors, multiple linear regression (MLR), and mixed integer linear programming (MILP). Selected descriptors based on a forward stepwise procedure enabled the simplest regression model, MLR, to achieve significantly good prediction accuracy compared to the existing approaches, achieving accuracy in the range [0.7191, 0.9377] for 29 diverse datasets. By simulating these descriptors and learning models as MILPs, we inferred mathematically exact and optimal compounds with the desired AS, prescribed structures, and up to 50 non-hydrogen atoms in a reasonable time range [6, 1166] seconds. These findings indicate a strong correlation between the simple graph-theoretic descriptors and the AS of compounds, potentially leading to a deeper understanding of their AS without relying on widely used complicated chemical descriptors and complex machine learning models that are computationally expensive, and therefore difficult to use for inference. An implementation of the proposed approach is available at https://github.com/ku-dml/mol-infer/tree/master/AqSol.

## Scientific contribution

We provide a thorough survey of prediction models designed for AS. Based on simple graph-theoretic descriptors, MLR, and MILP, we successfully predicted and inferred optimal compounds with the desired AS across diverse datasets. These findings indicate a strong correlation between the simple graph-theoretic descriptors and the AS of compounds, potentially leading to a deeper understanding of their AS without relying on widely used complicated chemical descriptors and complex machine learning models that are computationally expensive, and therefore difficult to use for inference.

## Introduction

The study of quantitative structure-activity/property relationship (QSAR/QSPR) and inverse QSAR/QSPR is crucial in the field of computational chemistry, bio-informatics and material informatics to understand the complex relationships between molecular structures and their properties [1]. QSAR/QSPR aims to predict the properties of a given chemical compound, while inverse QSAR/QSPR seeks to infer chemical compounds of desired properties.

Aqueous solubility (AS) of chemical compounds is a physiochemical property with great significance in various areas such as drug discovery, and material design [2]. There has been a notable focus on QSPR for the accurate prediction of AS through machine learning models such as multiple linear regression (MLR), logistic regression (LR), least absolute shrinkage and selection operator (LASSO), partial least square (PLS), and random forest (RF). We give a brief review of some recent prediction models as follows. Palmer et al. [3] developed a prediction model based on RF with 2D and 3D descriptors generated by molecular operating environment (MOE). In this study, one dataset was used for testing of the model, which may not be sufficient for an in-depth analysis. In addition, the 3D descriptors can be computationally expensive, and can be difficult to use in the inverse QSAR/QSPR (see Table 1). Raevsky et al. [4] represented chemical compounds by the descriptors generated from Hybot, Dragon, and VolSurf, and compared the accuracy of prediction models constructed by MLR, RF and support vector machine (SVM). They tested their models on one dataset, and the use of non-deterministic descriptors may limit the application of their model to the inverse QSAR/QSPR. Lowe et al. [5] utilized the PaDEL-Caret package to generate descriptors, and predicted aqueous solubility with RF. Similarly, testing was performed on only one dataset, and the descriptors were non-deterministic. Lovrić et al. [6] used LASSO, RF, and light gradient boosting machine (lightGBM). For the representation of compounds, they used fingerprints and non-deterministic molecular descriptors generated by Dragon. The models were tested on only one dataset. Tayyebi et al. [7] used MLR and RF with Mordred package to generate 2D and 3D descriptors and tested the models on one dataset. Wang et al. [8] employed MLR to construct prediction models by using 2D and 3D descriptors generated by Sybyl and Amber, respectively. In this study, the model was tested on five datasets with a minimum evaluation score of 0.4, which is well below the acceptable level. Meftahi et al. [9] generated diverse descriptors using Gauusian09, Sybyl, and BioPPSy to predict AS by MLR. They tested their model on seven datasets with a minimum evaluation score of 0.47, which may limits the application of their model to other datasets. Cao et al. [10] used PLS and advanced machine learning tools, back-propagation network (BPN) and support vector regression (SVR), to model the relationship between molecular descriptors and AS. These models were tested on one dataset with a minimum evaluation score 0.69 which is below the acceptable level. Deng et al. [11] used different neural networks such as convolution neural network (CNN), recurrent neural network (RNN), deep neural network (DNN), and shallow neural network (SNN) with non-deterministic molecular descriptors obtained from Dragon. Panapitiya et al. [12] used RDKit, Mordred, and Pybel for generating non-deterministic 2D and 3D descriptors and employed a graph neural network (GNN) for prediction. They tested their model on only one dataset. Hou et al. [13] proposed a deep learning model named bidirectional long short-term memory with channel and spatial attention network (BCSA) to generate non-deterministic descriptors and construct prediction models. They tested the model on five datasets and attained good evaluation scores. However, BCSA is computationally expensive and can be difficult to use in inverse QSAR/QSPR. Francoeur et al. [14] presented STN, a molecule attention transformer to predict aqueous solubility. They used SMILES representations of molecules and RDKit to generate descriptors. The model was tested on five datasets with a minimum evaluation score 0.65 less than the desired threshold. Moreover attention transformers are computationally expensive. Graph convolution neural networks (GCNN) were utilized by Conn et al. [15]. They used 2D and 3D non-deterministic descriptors generated by RDKit and Mordred, and tested the model on only one dataset. Li et al. [16] developed a model by using cuckoo search algorithm with light gradient boosting machine (CS-LightGBM) where molecular fingerprints were used as molecular representation to express the structure of compounds. The model was tested on one dataset. Tang et al. [17] introduced a self-attention-based message-passing neural network (SAMPN) model. They generated specific descriptors by message passing network encoder (MPN), and tested the model on a single dataset. A summary of these models is given in Table 1 with the number of testing datasets, descriptor information, software used to generate descriptors, and the evaluation scores $\text{R}^{2}$, where the minimum and maximum scores are listed if more than one dataset is used in the corresponding model.

**Table 1:** A summary of recent models used to predict aqueous solubility

S. no	Model	# datasets	Descriptor information (size)	Software	$\text{R}^2$ Min, Max
1	RF [3]	1	Deterministic 2D, 3D (200)	MOE	0.89
2	MLR, RF, SVM [4]	1	Non-deterministic (21)	Hybot, Dragon, Sybyl, VolSurf	0.701, 0.736
3	RF [5]	1	Non-deterministic (16)	PaDEL-Caret package	0.82
4	LASSO, PLS, RF, LightGBM [6]	1	Non-deterministic (317)	Dragon	N/A
5	MLR, RF [7]	1	Deterministic 2D, 3D	Mordred package	0.80, 0.98
6	MLR [8]	5	Deterministic 2D, 3D (58)	Sybyl, Amber	0.4, 0.9
7	MLR [9]	7	Deterministic (2, 3, 8)^a^	Gaussian09 program, Sybyl, BioPPSy	0.47, 0.87
8	PLS, BPN, SVR [10]	1	Deterministic (28)	Dragon	0.69, 0.735
9	CNN, RNN, DNN, SNN [11]	N/A	Non-deterministic (N/A)	N/A	N/A
10	GNN [12]	1	Non-deterministic 2D, 3D (839)	Mordred, Pybel, RDKit	0.76
11	BCSA [13]	5	Non-deterministic	Within model	0.83, 0.88
12	STN [14]	5	Deterministic (25)	RDKit	0.65, 0.89
13	GCNN [15]	1	Non-deterministic 2D, 3D (839)	Mordred, Pybel, RDKit	0.86
14	CS-LightGBM [16]	1	Non-deterministic	RDKit	0.8575
15	SAMPN [17]	1	Deterministic	MPN	N/A

^a^Different numbers of descriptors generated by different software

From Table 1, we can observe that most of the models are tested on a single dataset and a few are tested on five or seven datasets, which is very limited size for an in-depth analysis of a prediction model; some models used non-deterministic 3D and chemical descriptors, making it difficult to use them for the inverse QSAR/QSPR; and some of the listed models did not achieve good evaluation scores for all the tested datasets, thereby making their applicability to other datasets questionable. Furthermore, to the best of our knowledge, no inverse QSAR/QSPR model exists that is specifically designed to infer chemical compounds with the desired AS. These limitations of the existing models necessitate the exploration of simple deterministic descriptors and simple machine learning models to achieve high accuracy, thus allowing their efficient use in inverse QSAR/QSPR.

Recently, Azam et al. [18] proposed a novel framework based on machine learning models and MILP to infer acyclic chemical structures with a desired property value. Shi et al. [19] and Zhu et al. [20] extended this framework to infer chemical structures with rings. Similarly, Ido et al. [21] extended the framework for polymers. The framework has two phases: prediction phase and inference phase. A chemical compound is modeled as a chemical graph. Instead of using complicated non-deterministic chemical descriptors that are difficult to compute, and hence challenging for inverse QSPR, simple deterministic graph-theoretic descriptors are developed to construct prediction functions in the prediction phase. Other existing inverse QSPR approaches based on heuristics or statistical optimization algorithms do not ensure the exactness and optimality of the inferred chemical compounds, i.e., such approaches can infer invalid compounds, and the inferred compounds may not attain the desired property value. To avoid such issues in the inference phase of the framework, the descriptors and prediction functions are simulated by MILP formulations that are feasible if and only if there exists a desired chemical graph, and thus ensures the exactness and optimality of the inferred chemical graph. This formulation also allows the users to specify a prescribed structure to be preserved in the inferred graph.

Motivated by the importance of AS in drug discovery and material design, we aim to develop an approach that can address the shortcomings of the existing models. For this purpose, we use the framework [19, 20] to: (i) accurately predict AS for diverse datasets; and (ii) efficiently infer mathematically exact and optimal chemical compounds with the desired AS. The efficiency of this framework highly relies on the accuracy of the prediction phase. Therefore we modify the framework by introducing (a) a forward stepwise procedure (FSP) with MLR to select significant descriptors which are crucial for achieving high accuracy; and (b) different prediction strategies based on the simplest regression model, MLR, to construct good prediction functions. In contrast to the existing approaches, which are tested on a very limited number of datasets, we collected 29 diverse datasets to demonstrate the usefulness of our proposed approach. The main advantages of the proposed approach include the simple deterministic graph-theoretic descriptors that are computationally efficient to compute and a straightforward machine learning model, MLR, which is efficient to train. Despite its simplicity, the approach demonstrated the ability to construct accurate predictive functions for AS across all 29 datasets, achieving higher accuracy than recent methods based on advanced machine learning tools, such as artificial neural network (ANN), for several datasets. Additionally, the approach can infer chemical compounds with desired AS while ensuring the validity and optimality of the inferred compounds. It also allows users to specify a prescribed structure to be preserved in the inferred compounds. Computational experiments confirmed that the proposed approach successfully inferred several chemical graphs with desired AS and prescribed structures in a reasonable time. All datasets, source codes, and results are publicly available at https://github.com/ku-dml/mol-infer/tree/master/AqSol.

## Our approach

**Fig. 1:**
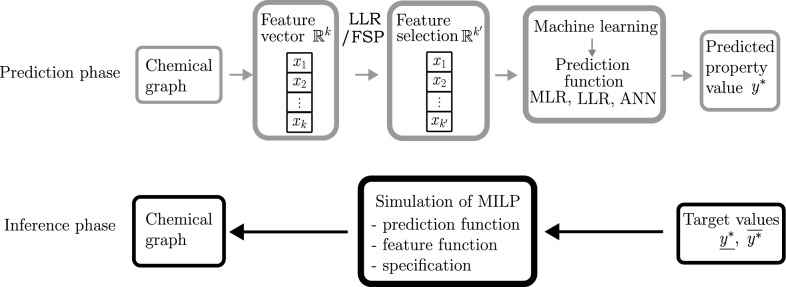
An illustration of our approach to inferring a chemical graph with the desired AS

Our approach is based on the framework [19, 20] to predict and infer chemical graphs with the desired AS. The inference phase of the framework highly depends on the learning performance of the prediction function constructed in the prediction phase. Therefore we modify the framework by introducing an FSP with MLR to select a set of best descriptors, and different learning strategies based on MLR to construct good prediction functions. The details of our approach are discussed in Sects. Prediction Phase and Inference Phase. An illustration of the approach is given in Fig. 1.

### Prediction phase

**Fig. 2:**
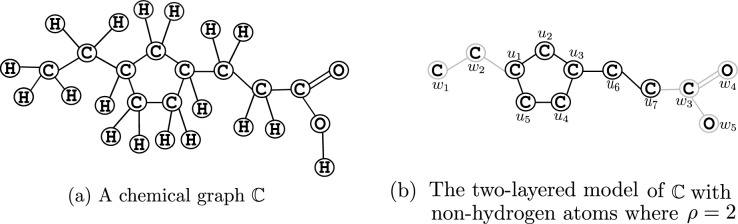
**a** Representation of the chemical compound 3-(3-Ethylcyclopentyl) propanoic acid with CID = 20849290 as a chemical graph ${\mathbb {C}}$; **b** The vertices and edges of the interior and exterior parts of ${\mathbb {C}}$ depicted with black and gray colors, respectively, in the two-layered model. The sets of interior and exterior vertices are $\{u_1, u_2, \ldots , u_7\}$ and $\{w_1, w_2, \ldots , w_5\}$, respectively

Modeling: We represent a chemical compound as a chemical graph based on the modeling introduced by Zhu et al. [20]. A chemical graph ${\mathbb {C}}=(H,\alpha ,\beta )$ consists of a simple connected and undirected graph *H*, a vertex-labeling $\alpha$ that keeps the information of chemical elements, such as C (carbon), O (oxygen), H (hydrogen) and N (nitrogen), at each vertex, and an edge-labeling $\beta$ that keeps the information of single, double, and triple bonds between two adjacent atoms. The chemical graph ${\mathbb {C}}$ of the compound 3-(3-Ethylcyclopentyl) propanoic acid is illustrated in Fig. 2a.

**Fig. 3:**
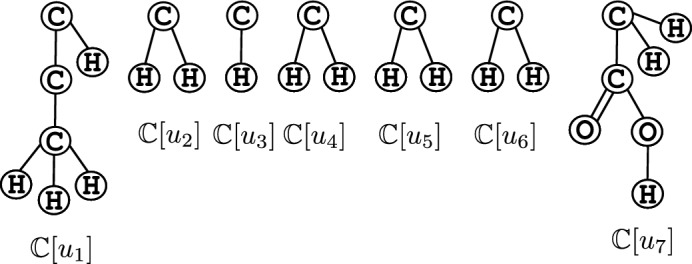
The 2-fringe trees ${\mathbb {C}}[u_i]$, $i \in [1,7]$ of the example ${\mathbb {C}}$ in Fig. 2a rooted at $u_i$

A chemical graph is divided into *interior part* and *exterior part* based on the two-layered model by Shi et al. [19]. For a given parameter $\rho$, the exterior part consists of non-root vertices and edges of rooted tree-like chemical subgraphs called $\rho$*-fringe trees* of height at most $\rho$. Intuitively, the fringe trees resemble terminal functional groups, which play an important role in the properties of the compounds. The subgraph other than the exterior part of a chemical graph is called the interior part (refer to Appendix A for details). The interior and exterior parts of the chemical graph given in Fig. 2a are depicted in Fig. 2b, where hydrogen atoms are ignored. The 2-fringe trees are illustrated in Fig. 3.

**Table 2:** Descriptors for the chemical graph ${\mathbb {C}}$ given in Fig. 2a

Descriptor	Descriptor value
Number of non-hydrogen atoms in ${\mathbb {C}}$	12
Rank of ${\mathbb {C}}$	1
Number of vertices in the interior	7
Average mass	56.667
Number of non-hydrogen vertices of degree 1	0
Number of non-hydrogen vertices of degree 2	5
Number of non-hydrogen vertices of degree 3	2
Number of non-hydrogen vertices of degree 4	0
Number of vertices of degree 1 in the interior	1
Number of vertices of degree 2 in the interior	5
Number of vertices of degree 3 in the interior	1
Number of vertices of degree 4 in the interior	0
Number of edges of bond multiplicity 2 in the interior	0
Number of edges of bond multiplicity 3 in the interior	0
Frequency of chemical elements in the interior:	
C	7
Frequency of chemical elements in the exterior:	
C	3
O	2
Frequency of edge-configurations in the interior:	
C 2 C 2 1	2
C 2 C 3 1	5
Frequency of fringe-configurations in the set of $\rho$-fringe trees:	
C 0 H 1	1
C 0 H 1 H 1	4
C 0 H 1 H 1 C 1 O 2 H 3	1
C 0 H 1 C 1 H 2 C 2 H 3 H 3	1
Frequency of adjacency-configurations in the set of leaf-edges:	
C C 1	1
O C 1	1
O C 2	1

Descriptors and their selection: Instead of using some complex chemical descriptors which are hard to compute and use in the inverse QSPR, we use simple and effective graph-theoretic descriptors introduced by Zhu et al. [20]. For a chemical graph ${\mathbb {C}}=(H,\alpha ,\beta )$, these descriptors are: the number of non-hydrogen atoms in ${\mathbb {C}}$; the rank of ${\mathbb {C}}$; the number of vertices in the interior; the average of mass over all atoms in ${\mathbb {C}}$; the number of vertices of degree $d, d\in \{1, 2,3, 4\}$ in ${\mathbb {C}}$ ignoring the vertices with hydrogen; the number of vertices of degree $d, d\in \{1, 2,3, 4\}$ in the interior ignoring the vertices with hydrogen; the number of edges with bond multiplicity *m*, $m\in \{2,3\}$ in the interior; the frequency of chemical elements in the interior; the frequency of chemical elements in the exterior; the frequency of *edge-configurations* in the interior which are defined to be the triplets (a*d*, b$d'$,*m*) for each edge $e = uv$ in the interior with $\alpha (u)=$ a, $\alpha (v)=$ b, degree of *u* (resp., *v*) equals to *d* (resp., $d'$) and $\beta (e)= m$; the frequency of *fringe-configurations* in the set of ${\rho }$-fringe-trees in ${\mathbb {C}}$; and the frequency of *adjacency-configurations* $(\text{a}, \text{b}, m)$ in the set of leaf-edges $e = uv$ with either *u* or *v* has degree 1 in ${\mathbb {C}}$, where $\alpha (u)=$ a, $\alpha (v)=$ b and $\beta (e)= m$. These descriptors are listed in Table 2 for an example chemical graph ${\mathbb {C}}$ given in Fig. 2a.

Selection of significant descriptors plays a key role in constructing good prediction functions. We introduce a descriptor selection method based on the forward stepwise procedure (FSP) [22] and MLR. FSP selects significant descriptors iteratively. That is, it starts with an empty set of selected descriptors, at each iteration adds a new descriptor from the set of unselected descriptors that has the optimal MLR evaluation score when combined with the current set of selected descriptors, and terminates the procedure when a desired number of descriptors is selected (refer to Appendix B for details). We also use LASSO linear regression (LLR) for descriptor selection in our approach.

Prediction strategies: We introduce different prediction strategies by using FSP for descriptor selection, MLR for prediction, and evaluation methods. These evaluation methods mainly depend on leave-one-out validation (LOOV) and cross validation (CV) (refer to Appendix C for details). The proposed prediction strategies are listed below:

- FSP-MLR: FSP is utilized to identify best descriptors, followed by the construction of a prediction function using MLR, and is evaluated by 10 times 5-fold CV.
- FSP-MLR-LOO: FSP is applied for selecting best descriptors with 5-fold CV for evaluation. Then MLR is employed for prediction, and the performance is evaluated using LOOV.
- FSP-LOO-MLR: FSP is used for the selection process and MLR is used for the prediction process. Both processes are evaluated by using LOOV.

Similarly, we also tried some other prediction strategies based on MLR, LLR and ANN. These strategies are listed below:

- MLR: MLR is applied without selecting descriptors with 10 times 5-fold CV for evaluation.
- MLR-LOO: MLR is applied without selecting descriptors utilizing LOOV.
- LLR-ANN: LASSO is used to identify best descriptors, followed by the construction of a prediction function using ANN. This strategy is evaluated by 10 times 5-fold CV. For more details, we refer to [23].
- LLR-ANN-LOO: LASSO is utilized to identify best descriptors followed by the construction of a prediction function using ANN which is evaluated by LOOV. This strategy is basically a modification of LLR-ANN [23].
- LLR-LLR: LASSO is utilized for selection of best descriptors and construction of a prediction function. The performance is evaluated by 10 times 5-fold CV. For more details, we refer to [20].

For all these strategies, we use the graph-theoretic descriptors.

### Inference phase

Several inverse QSPR models are available in the literature. However, most of these models heavily rely on heuristic algorithms or statistical optimization techniques, which often result in the inference of invalid compounds or compounds that do not attain the desired property value, and hence can be quite computationally expensive. In order to avoid such situations, we simulate the computation process of a prediction function by an MILP formulation due to Zhu et al. [20] to infer chemical graphs with the desired AS. A key advantage of this formulation is that it is feasible if and only if a desired chemical graph exists, implying that the inferred graphs will always be valid and achieve the desired AS. Furthermore, this formulation allows users to specify an abstract structure that is preserved in the inferred graph by using a *topological specification*. A topological specification is described as a set of following rules:

(i) a seed graph $G_{\mathbb {C}}$ that represents an abstract form of a target chemical graph ${\mathbb {C}}$;
(ii) a set $\mathcal {F}$ of chemical rooted trees that are selected for a tree ${\mathbb {C}}[u]$ with root at each vertex *u* in the exterior; and
(iii) lower and upper bounds for the number of components such as vertices in the interior and double/triple bonds within a target chemical graph ${\mathbb {C}}$.

**Fig. 4:**
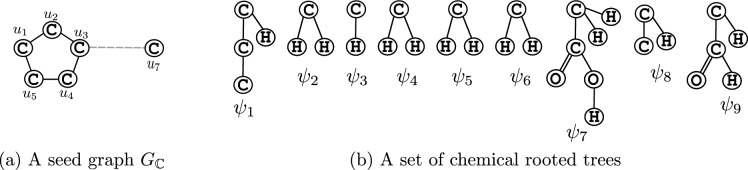
**a** An illustration of seed graph $G_{\mathbb {C}}$ for chemical graph given in Fig. 2a with a typical edge depicted by a dashed line; **b** A set $\mathcal {F} = \{\psi _1, \psi _2, \ldots , \psi _9\}$ of chemical rooted trees, where hydrogen atoms with non-root vertex are omitted

For a given seed graph $G_{{\mathbb {C}}}$, the formulation constructs the interior of a target chemical graph ${\mathbb {C}}$ by replacing typical edges with paths, and exterior by attaching fringe trees. Figures 4a, 4b illustrate an example of a seed graph $G_{\mathbb {C}}$ with a typical edge, and a set $\mathcal {F}$ of chemical rooted trees, respectively. The chemical graph given in Fig. 2a can be obtained from the seed graph $G_{\mathbb {C}}$ by replacing the typical edge with a path of length 2, and then attaching the fringe trees from $\mathcal {F}$ accordingly.

## Experimental results and discussion

We implemented and tested the proposed approach on a PC with Processor: Core i3 (2.6 GHz at the maximum) and Memory: 6 GB RAM. In contrast to the existing approaches that are tested on a very limited number of datasets, we collected 29 diverse datasets to demonstrate the usefulness of our approach.

**Table 3:** Summary of datasets

$\mathcal {C}$	$|\mathcal {C}|$	$\underline{y},~\overline{y}$	|*D*|
Protac	21	$-6.64,\,-3.18$	83
Wassvik	26	$-8.49,\,-2.48$	94
Alex Manfred	72	$-0.833,\,0.65$	163
Goodman	87	$-6.74,\,-1.06$	130
D5	91	$-5.88,\,0.58$	118
Duffy	98	$-10.32,\,-2.48$	139
Boobier	99	$-8.8,\,1.7$	133
Dearden	118	$-6.24,\,-0.57$	142
Ran	129	$-10.8,\,2.06$	157
Llinas	132	$-8.75,\,-1.18$	167
Bergstrom	163	$-7.59,\,0.55$	154
Grigorev	362	$-7.85,\,0.38$	173
Jain	456	$-12.95,\,1.58$	223
Lovric	805	$-8.75,\,1.149$	323
Huuskonen	827	$-11.62,\,1.58$	310
David	826	$-10.41,\,1.58$	263
Water set wide	845	$-12.79,\,1.58$	320
Daniel	915	$-10.43,\,6.4$	372
Esol	1054	$-11.6,\,1.58$	338
Aqua	1238	$-11.62,\,1.58$	364
Tang	1221	$-1162,\,1.58$	364
Wang	1414	$-9.33,\,1.58$	405
Phys	1812	$-12.06,\,1.58$	469
Training set	5315	$-13.17,\,2.89$	675
Ochem	6006	$-12.1,\,1.58$	668
Cui	6678	$-18.21,\,1.7$	766
Aqsol	8230	$-13.17,\,2.13$	965
Charles N. Lowe	*9150	$-13.17,\,2.41$	835
Ademola	10343	$-13.17,\,2.14$	949

$\mathcal {C}$: the dataset; $|\mathcal {C}|$: the size of $\mathcal {C}$ after the preprocessing; *the preprocessing is performed on 10000 randomly selected chemical compounds; $\underline{y},~\overline{y}$: the lower and upper bounds of AS in each dataset; and |*D*|: the total number of descriptors

Datasets: The 29 diverse datasets are: Protac, Alex Manfred, Ran Yalkowsky, Llinas, Water set wide [24], Wassvik, Duffy, Dearden, Huuskonen [9], D5, Jain, Goodman, Wang [8], Boobier, Aqsol, ESOL [14], Bergstrom [25], Grigorev [4], Lovric [6], David [3], Daniel [26], Tang [12], Phys, Ochem, Aqua  [27], Training set [15], Cui [13], Charles N. Lowe [5], and Ademola [28].

Preprocessing: As a preprocessing, some chemical compounds that do not satisfy one of the following conditions are removed: the graph is connected, the number of carbon atoms is at least four, and the number of non-hydrogen neighbors of each atom is at most 4. The compounds that are not available in PubChem database [29] are also removed. A summary of the datasets is given in Table 3. These datasets have size in the range [21, 10343], AS values in the range $[-13.17, 2.14]$, and the number of graph-theoretic descriptors in the range [83, 965].

### Results on prediction phase

Prediction functions are constructed for the 29 datasets based on the prediction strategies by using Python 3.11.3 and Scikit-learn version 1.2.2.

Based on preliminary experiments, the strategies with LOOV are used for relatively small datasets, with a size of at most 150. For such 11 datasets, the $\text{R}^2$ scores due to MLR-LOO, LLR-ANN-LOO, FSP-MLR-LOO, and FSP-LOO-MLR strageties are listed in Table 4. For the remaining 18 datasets, the results of the prediction strategies MLR, LLR-ANN, LLR-LLR, and FSP-MLR are listed in Table 5.

**Table 4:** $\text{R}^2$ scores for small datasets due to MLR-LOO, LLR-ANN-LOO, FSP-MLR-LOO and FSP-LOO-MLR

$\mathcal {C}$	$|D^*_\text{L}|$	$|D^*_\text{F}|$	$|D^*_\text{FL}|$	$\text{R}^2_{\text { MLR-LOO}}$	$\text{R}^2_{\text { LLR-ANN-LOO}}$ [23]	$\text{R}^2_{\text { FSP-MLR-LOO}}$	$\text{R}^2_{\text { FSP-LOO-MLR}}$	$\text{R}^2$ model
Protac	10	6	10	$-235.96$	0.7709	0.6728	0.7709	$-0.18$ [24] DeepNN
Wassvik	10	2	10	$-0.0125$	0.6780	0.6788	$\mathbf {0.8624}$	0.95 [9] MLR
D5	10	20	25	$-4 \text{E}10$	0.7389	0.2043	$^*\mathbf {0.8455}$	0.627 [8] MLR
Alex Manfred	13	10	30	0.044	0.6573	0.647	$^*\mathbf {0.7593}$	0.36 [24] RF
Goodman	18	40	20	$-8\text{E}10$	0.5147	$^*\mathbf {{0.7886}}$	0.6363	0.527 [8] MLR
Duffy	47	12	45	$-2\text{E}10$	0.7863	$-0.2177$	$\mathbf {0.9266}$	0.94 [9] MLR
Boobier	29	9	50	$-8.3\text{E}10$	0.6620	$-0.1734$	$^*\mathbf {0.8201}$	0.773 [14] STN
Dearden	43	10	55	$-6.6\text{E}9$	0.6987	$-0.0601$	$\mathbf {0.7191}$	0.87 [9] MLR
Ran	39	22	50	$-8\text{E}10$	0.6719	$^*\mathbf {0.8931}$	0.8041	0.82 [24] XGBoost
Llinas	25	10	35	$-2.3\text{E}10$	0.5175	$^*\mathbf {0.7853}$	0.6690	0.46 [24] XGBoost
Bergstrom	42	14	40	$-7.6\text{E}10$	0.7251	$-0.0267$	$^*\mathbf {0.8138}$	0.736 [4] RF

$|D^*_\text{L}|$: the number of descriptors selected in LLR-ANN-LOO; $|D^*_\text{F}|$: the number of descriptors selected in FSP-MLR-LOO; $|D^*_\text{FL}|$: the number of descriptors selected in FSP-LOO-MLR; $\text{R}^2_{\text { MLR-LOO}}$: the $\text{R}^2$ score of test data due to MLR-LOO; $\text{R}^2_{\text { LLR-ANN-LOO}}$: the $\text{R}^2$ score of test data due to LLR-ANN-LOO; $\text{R}^2_{\text { FSP-MLR-LOO}}$: the $\text{R}^2$ score of test data due to FSP-MLR-LOO; $\text{R}^2_{\text { FSP-LOO-MLR}}$: the $\text{R}^2$ score of test data due to FSP-LOO-MLR; $\text{R}^2$: the $\text{R}^2$ score of the existing model; N/A: results not available; bold score indicates the best score among our prediction strategies; and $^*$ indicates that our best score is better than the scores achieved by the existing models

**Table 5:** $\text{R}^2$ scores for larger datasets due to MLR, LLR-ANN, LLR-LLR, and FSP-MLR

$\mathcal {C}$	$|D^*|$	$|D^*_{\text { FSP-MLR}}|$	$\text{R}^2_\text{MLR}$	$\text{R}^2_{\text { LLR-ANN}}$ [23]	$\text{R}^2_{\text { LLR-LLR}}$ [20]	$\text{R}^2_{\text { FSP-MLR}}$	$\text{R}^2$ model	$\text {Time}_\text{FSP}$
Grigorev	81	42	$-2.34\text{E}23$	0.6685	0.6721	$\mathbf {0.7612}$	N/A	75
Jain	97	45	$-1.76\text{E}22$	0.9086	0.9312	$\mathbf {0.9377}$	0.943 [8] MLR	65
Lovric	66	35	$-6.815\text{E}22$	0.7079	0.7144	$\mathbf {0.7294}$	N/A	85
Huuskonen	141	50	$-1.64\text{E}22$	0.8167	0.8259	$\mathbf {0.8371}$	0.84 [9] MLR	105
David	105	43	$-7.21\text{E}21$	0.8410	$\mathbf {0.8521}$	0.8482	0.896 [3] RF	74
Water set wide	81	51	$-2.10\text{E}23$	$^*\mathbf {0.8195}$	0.7941	0.7975	0.77 [24] XGBoost	124
Daniel	149	40	$-7.899\text{E}24$	$\mathbf {0.8348}$	0.8114	0.8264	0.935 [26] SVM	105
Esol	222	60	$-7.14\text{E}21$	$\mathbf {0.8659}$	0.8147	0.8171	0.911 [14] STN	163
Aqua	138	45	$-4.75\text{E}23$	$\mathbf {0.8465}$	0.8270	0.8399	N/A	136
Tang	154	60	$-2.47\text{E}23$	$\mathbf {0.8487}$	0.8211	0.8307	N/A	142
Wang	145	47	$-5.76\text{E}23$	$\mathbf {0.8441}$	0.7485	0.7578	0.881 [8] MLR	159
Phys	130	60	$-3.64\text{E}23$	$\mathbf {0.8867}$	0.8287	0.8382	N/A	276
Training set	372	120	$-1.24\text{E}23$	$\mathbf {0.8369}$	0.7752	0.7776	0.86 [15] GCNN	3322
Ochem	469	110	$-4.98\text{E}22$	$\mathbf {0.9313}$	0.8405	0.8608	N/A	7067
Cui	114	80	$-9.05\text{E}23$	0.7803	0.7619	$\mathbf {0.7806}$	0.8813 [13] BCSA	1681
Aqsol	285	113	$-5.44\text{E}24$	$\mathbf {0.8184}$	0.7198	0.7185	N/A	6300
Charles N. Lowe	565	250	$-1.54\text{E}21$	$\mathbf {0.8849}$	0.7476	0.7957	0.97 [5] RF	70464
Ademola	97	180	$-1.07\text{E}24$	$\mathbf {0.8675}$	0.7498	0.7830	N/A	29308

$|D^*|$: the number of descriptors selected in the strategies LLR-ANN and LLR-LLR; $|D^*_{\text { FSP-MLR}}|$: the number of descriptors selected in FSP-MLR; $\text{R}^2_\text{MLR}$: the median of $\text{R}^2$ score of test data due to MLR; $\text{R}^2_{\text { LLR-ANN}}$: the median of $\text{R}^2$ score of test data due to LLR-ANN; $\text{R}^2_{\text { LLR-LLR}}$: the median of $\text{R}^2$ score of test data due to LLR-LLR; $\text{R}^2_{\text { FSP-MLR}}$: the median of $\text{R}^2$ score of test data due to FSP-MLR; $\text{R}^2$: $\text{R}^2$ score of the existing model; N/A: results not available; $\text{Time}_\text{FSP}$: running time, in seconds, to obtain reduced descriptors by using FSP; $a \text{E} b$ represents $a\!\times \! 10^{b}$; bold score indicates the best score among our prediction strategies; and $^*$ indicates that our best score is better than the scores achieved by the existing models

From Tables 4 and 5, the performance of MLR alone is poor. However our FSP-MLR-based strategies in which descriptors are selected by FSP and then MLR is applied greatly improved the results. Specifically, the best R$^2$ score among the FSP-MLR-based strategies for each of the 29 datasets is at least 0.7198, which falls within the acceptable range, confirming the effectiveness of the proposed strategies. Similarly, our strategies significantly outperform (resp., yield comparable results to) the strategies based on LLR and ANN in [20, 23] for relatively small (resp., large) datasets. Notably, FSP-MLR-based strategies achieved the best scores on 16 datasets. Additionally, our strategies outperform existing results for nine datasets, particularly improving scores for the datasets such as Protac, D5, Alex Manfred, Goodman, and Llinas from $-0.18$, 0.625, 0.36, 0.527, and 0.46 to 0.8769, 0.8455, 0.7593, 0.7830, and 0.7853, respectively. For the remaining 13 datasets with available scores, the results are comparable. These good evaluation scores are achieved by selecting a small number of descriptors. For the small (resp., large) datasets, our model selected descriptors in the range $[6, 39](\%)$ (resp., $[10, 16](\%)$) with an average 21% (resp., 16%), which are significantly smaller than those selected by LLR [23] in the range $[10, 70](\%)$ with an average 40%. In our experiments, we trained the MLR and ANN models on the same datasets for a fair comparison. The computational time of ANN is much bigger than MLR, therefore we restricted the size of the dataset to 10,343. The running time for FSP to reduce the descriptors is in the range of [65, 70464] seconds. From Table 5, we observe that the running time for FSP increases with the number of chemical compounds and selected descriptors.

These experimental results demonstrate that the small numbers of selected graph-theoretic descriptors enabled the simplest regression model MLR to achieve good evaluation scores across the diverse datasets. This indicates a strong correlation between graph-theoretic descriptors and the AS of chemical compounds, paving the way to understanding AS without relying on widely used 3D and chemical descriptors and complex machine learning models, which can be computationally expensive.

### Results on inference phase

We selected the datasets Jain and Duffy (resp., Wang and Phys) for which FSP-MLR (resp., LLR-ANN) constructed prediction functions with relatively higher evaluation scores. For an in-depth analysis, we prepare seven different instances namely $I_{\text{a}}$, $I_{\text{b}}^i, i\in \{1, 2, 3, 4\}$, $I_{\text{c}}$ and $I_{\text{d}}$ with carefully crafted different seed graphs developed by Zhu et al. [20]. The seed graph of instance $I_{\text{a}}$ is designed to infer any prescribed structures, whereas the seed graphs of instances $I_{\text{b}}^i, i\in \{1, 2, 3, 4\}$ are designed to infer chemical graphs of rank 1 or 2. The seed graphs of instances $I_{\text{c}}$ and $I_{\text{d}}$ are designed by merging the structural information of two chemical compounds obtained from PubChem database [29] to infer a chemical graph that somehow preserves the structure of the two chemical compounds. These instances also heavily depend on other specifications such as the set $\mathcal {F}$ of chemical rooted trees, lower and upper limits for the frequency of chemical symbols, edge configurations and adjacency configurations. We fixed these specifications according to each of the four selected datasets Duffy, Jain, Wang, and Phys. MILP formulations are solved by using CPLEX version 22.1.1. Tables 6 and 7 (resp., Tables 8 and 9) show the experimental results of the inference phase for the datasets Jain and Duffy (resp., Wang and Phys).

**Table 6:** Results of the inference phase for the dataset Jain

Inst.	$n_{\text{LB}}$	$\underline{y^*},~\overline{y^*}$	$\#$v	$\#$c	I-time	*n*	$\eta (f({\mathbb {C}}^{\dagger }))$
$I_{\text{a}}$	30	$-18.75, -18.7$	10535	9034	30.787	49	$-18.702$
$I_{\text{b}}^1$	35	$-12.5, -12.45$	10402	6680	11.333	35	$-12.47$
$I_{\text{b}}^2$	45	$-9.95, -9.9$	13123	9802	58.809	48	$-9.903$
$I_{\text{b}}^3$	45	$-13.95, -13.9$	12913	9804	177.04	50	$-13.907$
$I_{\text{b}}^4$	45	$-3.9, -3.85$	12707	9810	110.082	50	$-3.854$
$I_{\text{c}}$	50	$-9.2, -9.15$	6651	6980	6.583	50	$-9.158$
$I_{\text{d}}$	40	$-9.7, -9.65$	5271	6479	67.799	44	$-9.699$

$n_{\text{LB}}$: lower bound for the number of non-hydrogen atoms of target graph ${\mathbb {C}}$; $\underline{y^*},~\overline{y^*}$: lower and upper limits $\underline{y^*},~\overline{y^*}\in {\mathbb {R}}$ on the AS of a target graph ${\mathbb {C}}$; $\#$v and $\#$c are the number of variables and constraints in the MILP, respectively; I-time: the MILP solution time (sec.); *n*: the number of non-hydrogen atoms; and $\eta$: the predicted property value $\eta (f({\mathbb {C}}^{\dagger }))$ of the inferred chemical graph ${\mathbb {C}}^{\dagger }$

**Table 7:** Results of the inference phase for the dataset Duffy

Inst.	$n_{\text{LB}}$	$\underline{y^*},~\overline{y^*}$	$\#$v	$\#$c	I-time	*n*	$\eta (f({\mathbb {C}}^{\dagger }))$
$I_{\text{a}}$	30	$-11.5, -11.45$	10535	9035	16.646	42	$-11.498$
$$I_{\text{b}}^1$$	35	$$-10.9, -10.85 $$	10717	6647	12.458	35	$$-10.869$$
$I_{\text{b}}^2$	45	$-8.4, -8.35$	13536	9767	65.218	50	$-8.39$
$I_{\text{b}}^3$	45	$-3.95, -3.9$	13337	9773	31.167	50	$-3.936$
$I_{\text{b}}^4$	45	$-14.45, -14.4$	13138	9778	91.762	49	$-14.432$
$I_{\text{c}}$	50	$-10.1, -10.05$	6651	6981	6.328	50	$-10.054$
$I_{\text{d}}$	40	$-8.05, -8$	5271	6482	18.634	44	$-8.018$

**Table 8:** Results of the inference phase for the dataset Wang

Inst.	$n_{\text{LB}}$	$\underline{y^*},~\overline{y^*}$	$\#$v	$\#$c	I-time	*n*	$\eta (f({\mathbb {C}}^{\dagger }))$
$I_{\text{a}}$	30	$-6, -5$	11212	10504	25.871	35	$-5.911$
$I_{\text{b}}^1$	35	$-2.5, -2.45$	15015	8633	962.451	35	$-2.471$
$I_{\text{b}}^2$	45	$-2.8, -2.3$	18943	11747	338.681	50	$-2.35$
$I_{\text{b}}^3$	45	$-2, -1.5$	18784	11747	251.548	50	$-1.583$
$I_{\text{b}}^4$	45	$-2.8, -2.351$	18624	11746	751.557	50	$-2.3$
$I_{\text{c}}$	50	$-4.5, -3.5$	7319	8435	224.792	50	$-3.836$
$I_{\text{d}}$	40	$-5, -4$	5942	7940	13.319	42	$-4.475$

**Table 9:** Results of the inference phase for the dataset Phys

Inst.	$n_{\text{LB}}$	$\underline{y^*},~\overline{y^*}$	$\#$v	$\#$c	I-time	*n*	$\eta (f({\mathbb {C}}^{\dagger }))$
$I_{\text{a}}$	30	$-7.8, -7.75$	10880	9749	376.331	45	$-7.755$
$I_{\text{b}}^1$	35	0.15, 0.2	14467	7855	214.926	35	0.191
$I_{\text{b}}^2$	45	0.52, 0.57	18326	10970	1165.992	50	0.562
$$I_{\text{b}}^3$$	45	$$-3.6, -3.55 $$	18166	10971	322.07	50	$$-3.57$$
$I_{\text{b}}^4$	45	0.01, 0.06	18000	10968	276.396	45	0.029
$I_{\text{c}}$	50	$-5.47, -5.42$	6987	7680	10.644	50	$-5.436$
$I_{\text{d}}$	40	$-2.25, -2.2$	5610	7185	68.318	41	$-2.235$

**Fig. 5:**
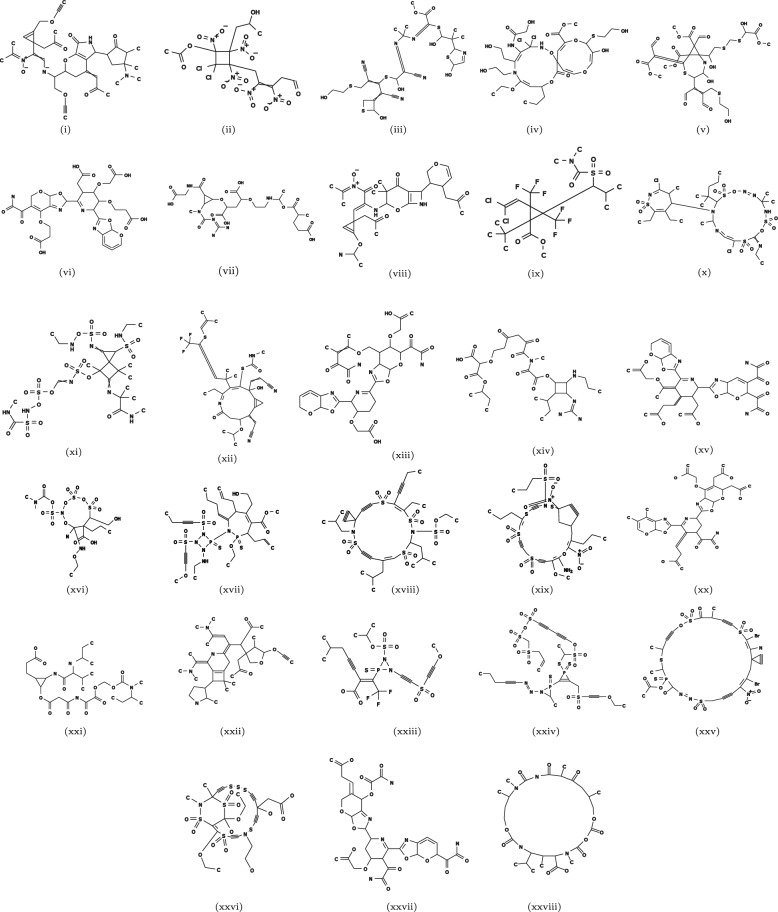
(i)-(vii), (viii)-(xiv), (xv)-(xxi), and (xxii)-(xxviii) inferred chemical graphs using the datasets Jain, Duffy, Wang and Phys, respectively

To validate the inferred chemical graph ${\mathbb {C}}^{\dagger }$, the AS $\eta (f({\mathbb {C}}^{\dagger }))$ is also predicted using the corresponding prediction function. The experimental results show that even with narrow desired ranges of the AS of the target graphs, the MILP formulations successfully inferred chemical graphs ${\mathbb {C}}^{\dagger }$ with AS $\eta (f({\mathbb {C}}^{\dagger }))$ within the desired ranges while preserving the prescribed structures, confirming the effectiveness of the MILP formulation. Additionally, the MILP formulations inferred graphs with relatively larger sizes, with the number of non-hydrogen atoms ranging from 35 to 50, within a reasonable time frame [6.328, 1165.992] seconds, demonstrating the efficiency of the inference phase. It is observed that the instances with a large number of variables and constraints may require more time compared to the instances with fewer variables and constraints. Furthermore, the MILP solution time also depends on the complexity of the instances. That is, a complex instance with fewer variables and constraints can take more time as compared to a relatively simpler instance with more variables and constraints. For example, the running time to solve instance $I_\text{c}$ is smaller than that of instance $I_\text{d}$, even though the number of variables and constraints in $I_\text{c}$ is larger than in $I_\text{d}$. It is also observed that the MILP solution time when using MLR is significantly shorter than when using ANN. For example, the solution time for instance $I_{\text{b}}^2$ ranges from 11 to 12 s with MLR, compared to 339 to 1166 s with ANN. This difference can be due to the lower complexity of the prediction function constructed by MLR as compared to that of ANN. All the inferred chemical graphs are illustrated in Fig. 5.

## Conclusion

A unified approach is proposed to predict and infer chemical compounds with the desired AS. Prediction is performed by modeling a chemical compound as a chemical graph with interior and exterior parts which are represented as graph-theoretic descriptors. FSP is used to extract significant descriptors followed by MLR to construct prediction functions. Graph-theoretic descriptors and prediction functions are simulated by MILPs to infer mathematically exact and optimal chemical graphs with the desired AS and prescribed structure.

For an in-depth analysis, the proposed FSP-MLR-based prediction strategy was tested on 29 diverse datasets and achieved acceptable evaluation scores for all of the datasets. Our strategies attained significantly higher evaluation scores compared to the recent existing scores, especially improving the scores for the datasets Protac, D5, Alex Manfred, Goodman, and Llinas from $-0.18$, 0.625, 0.36, 0.527, and 0.46 to 0.8769, 0.8455, 0.7593, 0.7830, and 0.7853, respectively. Several chemical graphs with up to 50 non-hydrogen atoms were successfully inferred with the desired AS and prescribed structures for different datasets in a reasonable computation time ranging from 6 to 1166 s. Furthermore, the MILP formulation with MLR-based prediction functions has significantly lower computation time than with ANN-based functions. This confirms the effectiveness of our simple approach without relying on complex machine learning models which are quite computationally expensive.

Experimental results show that the small number of selected graph-theoretic descriptors enabled the simplest regression model, MLR, to achieve high evaluation scores across the diverse datasets, indicating a strong correlation between these descriptors and the AS of chemical compounds. Future work will focus on exploring and investigating the relationships between graph-theoretic descriptors and the AS of compounds that result in a strong correlation, aiming for a better understanding of AS without relying on complicated non-deterministic chemical descriptors. In our experiments, we used datasets ranging in size from 21 to 10343, for which FSP efficiently extracted descriptors. However, due to the exhaustive search in FSP, descriptor extraction can be computationally expensive for large datasets. Furthermore, it was observed that the computation time of the MILP formulation depends on the complexity of the underlying data as well as the number of variables and constraints. Therefore, future work will focus on efficiently handling large datasets and inferring larger and more complex chemical graphs.

## Figures and Tables

**Algorithm 1:**
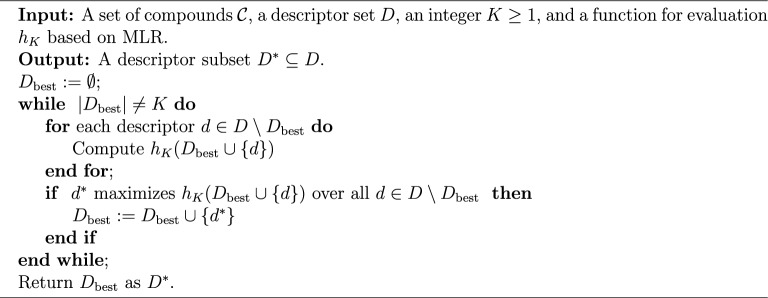
Forward stepwise procedure with MLR

## Data Availability

All datasets, source codes and results are available at https://github.com/ku-dml/mol-infer/tree/master/AqSol.

## Appendix

In the following sections, we denote by *V*(*G*) and *E*(*G*), the vertex set and edge set, respectively, of a graph *G*. A path with two end-vertices *u* and *v* is denoted by *u*, *v**-path*. The *rank* of a graph is defined to be the minimum number of edges to be removed so that the graph has no cycles.

A *rooted* graph is defined to be a graph with a designated vertex, called a *root*. The *height* of a vertex in a rooted tree is defined to be the size of the longest path from that vertex to a leaf. The height of a rooted tree *T* is defined to be the maximum height of a vertex in *T*, and is denoted by $\text{ht}(T)$. A chemical compound ${\mathbb {C}}=(H,\alpha ,\beta )$, is represented by a simple connected and undirected graph *H* and functions $\alpha :V(H)\rightarrow \Lambda$ and $\beta : E(H)\rightarrow \{1, 2, 3\}$, where $\Lambda$ is a set of chemical elements such as C (carbon), O (oxygen), H (hydrogen) and N (nitrogen). Define the *hydrogen-suppressed chemical graph* $\langle \mathbb {C} \rangle$ of a chemical graph ${\mathbb {C}}$ to be the graph obtained from *H* by removing all the vertices whose label is H. Figure 2b illustrates an example of a hydrogen-suppressed chemical graph $\langle \mathbb {C} \rangle$ obtained from the chemical graph ${\mathbb {C}}$ of chemical compound 3-(3-Ethylcyclopentyl) propanoic acid given in Fig. 2a.

## Appendix A

Two-layered model

Functional groups are characteristic groups of atoms that are attached to chemical compounds to attain desired chemical properties. In other words, functional groups play an important role in determining the chemical properties of chemical compounds. Building on this observation, Shi et al. [19] proposed a novel two-layered model where a hydrogen-suppressed chemical graph is partitioned into interior and exterior parts to capture key information of chemical compounds. Intuitively, the interior part primarily consists of rings and the paths connecting them, while the exterior part includes tree-like structures attached to the interior. In this model, the exterior part resembles functional groups without rings, and its size is controlled by an external parameter. This partition allows capturing important details of chemical compounds that can be crucial for a machine learning model to learn a given property. Formally, let ${\mathbb {C}}=(H,\alpha ,\beta )$ be a chemical graph and $\rho$ be a positive integer.

The purpose of the parameter $\rho$ is to control the size of the exterior part in the two-layered model as follows.

A vertex $v\in V(\langle \mathbb {C} \rangle )$ (resp., an edge $e\in E(\langle \mathbb {C} \rangle ))$ of ${\mathbb {C}}$ is called an *exterior-vertex* (resp., *exterior-edge*) if *v* is a non-root vertex of a rooted tree with height at most $\rho$ (resp., *e* is incident to an exterior-vertex). We denote the sets of exterior-vertices and exterior-edges by $V^\text{ext}({\mathbb {C}})$ and $E^\text{ext}({\mathbb {C}})$, respectively.

Additionally, we define $V^\text{in}({\mathbb {C}})=V(\langle \mathbb {C} \rangle )\setminus V^\text{ext}({\mathbb {C}})$ and $E^\text{in}({\mathbb {C}})=E(\langle \mathbb {C} \rangle )\setminus E^\text{ext}({\mathbb {C}})$, to denote the sets of interior vertices and interior edges, respectively.

The set $E^\text{ext}({\mathbb {C}})$ of exterior-edges forms a collection of connected graphs each of which is considered as a rooted tree *T* rooted at the vertex $v\in V(T)$ with maximum $\text{ht}(v) \le \rho$. Let $\mathcal {T}^\text{ext}(\langle \mathbb {C} \rangle )$ denote the set of these chemical rooted trees in $\langle \mathbb {C} \rangle$. The *interior* ${\mathbb {C}}^\text{in}$ of ${\mathbb {C}}$ is defined to be the subgraph $(V^\text{in}({\mathbb {C}}),E^\text{in}({\mathbb {C}}))$ of $\langle \mathbb {C} \rangle$. For $\rho$ = 2, and the example $\langle \mathbb {C} \rangle$ given in Fig. 2(b), the interior can be obtained by iteratively removing the set of vertices with degree 1 two times, where $\{w_1, w_2, \ldots , w_5\}$ and $\{u_1, u_2, \ldots , u_7\}$. For each $u\in V^\text{in}({\mathbb {C}})$, let $T_u\in \mathcal {T}^\text{ext}(\langle \mathbb {C} \rangle )$ denote the chemical tree rooted at *u*, and we define the $\rho$-fringe tree to be the chemical rooted tree obtained from $T_u$ by putting back the hydrogens. These $\rho$-fringe trees resemble with functional groups. Figure 3 illustrates the set of 2-fringe trees of the chemical graph ${\mathbb {C}}$ in Fig. 2(a).

## Appendix B

Descriptor selection

Let $\mathcal {C}$ be a dataset of chemical graphs ${\mathbb {C}}$, and $a({\mathbb {C}})\in {\mathbb {R}}$ denote observed value of aqueous solubility of ${\mathbb {C}}$. Let *D* be a set of descriptors and *f* represents a feature function that assigns a vector $f({\mathbb {C}})=x \in {\mathbb {R}}^{|D|}$ to a graph ${\mathbb {C}}$. The value of descriptor $d\in D$ is denoted by *x*(*d*).

An algorithmic description of the descriptor selection method with FSP and MLR is given in Algorithm 1, where for a subset $D^*\subseteq D$, $\text{R}^2_{\text{MLR}}(\eta ,D^*)$ denote the $\text{R}^2$ score of a prediction function $\eta$ obtained by MLR using descriptor set $D^*$, and for an integer $K\ge 1$, $h_K:2^D\rightarrow {\mathbb {R}}$ is an evaluation function such that $h_K(D^*)=\text{R}^2_{\text{MLR}}(\eta ,D^*)$.

## Appendix C

Machine learning models and evaluation

We use linear regression and ANN to construct a prediction function which are briefly explained as follow.

Linear regression: It tries to identify the best linear relationship between the given data points and their observed values. More precisely, it finds a hyperplane that minimizes the error between the observed values and predicted values of the given dataset. LLR [30] uses an extra regularization term with the error function to penalize coefficients of the hyperplane. Thus LLR selects a hyperplane that minimizes the following LASSO function:

$$\frac{1}{2|\mathcal {C}|}\text{Err}(\eta _{w, b}; \mathcal {C}) + \lambda ( \sum _{j =1}^{|D|} |w(j)| + |b| ), $$

where $\mathcal {C}$ is the dataset of chemical graphs with $a_i$ and $x_i$ to be the observed value and feature vector, resp., of ${\mathbb {C}}_i \in \mathcal {C}$; *w*, *b* are the coefficients of the constructed hyperplane, $\eta _{w, b}$ is the prediction function due to the hyperplane *w*, *b*; $\lambda$ is the regularization term, and $\text{Err}(\eta ;\mathcal {C})$ is the error function such that

$$ \text{Err}(\eta _{w, b};\mathcal {C}) \triangleq \sum _{{\mathbb {C}}_i\in \mathcal {C}}(a_i - \eta _{w, b}(x_i))^2.$$

MLR can be considered as a special case of LASSO function when $\lambda = 0$.

Artificial neural network: An ANN consists of three kinds of layers: an input layer, hidden layers, and an output layer, with each layer consisting of nodes. There are weighted edges between every two consecutive layers and a bias term for each node. In this work, we use fully connected feed-forward ANN with rectification linear unit (ReLU) and identity function as activation functions in the hidden layers and output layer, resp., where $\text{ReLU}(x) \triangleq \text{max}(0, x).$ The input layer takes the input data, and the output layer provides the predicted value. At each node of the hidden and output layers, the computation of the weighted sum followed by the application of an activation function is performed. The ANN algorithm tries to update the weights and biases to minimize the error between observed and predicted values.

Evaluation: Let *K* denote that number of descriptors used. To evaluate the performance of prediction functions $\eta : {\mathbb {R}}^K\rightarrow {\mathbb {R}}$ constructed by our model we define *coefficient of determination* $\text{R}^{2}(\eta ;\mathcal {C})$ as

$$ \displaystyle { \text{R}^{2}(\eta ; \mathcal {C} ) \triangleq 1- \frac{\text{Err}(\eta ;\mathcal {C}) }{\sum _{ {\mathbb {C}}_i\in \mathcal {C} } (a_i-\widetilde{a})^2} \text{ for } \widetilde{a}= \frac{1}{|\mathcal {C}|}\sum _{ {\mathbb {C}}_i \in \mathcal {C} }a({\mathbb {C}}_i) }. $$

.

We perform 5-fold CV and LOOV as follows:

**1.**: 5-fold CV: A random partition is made for a set of graphs $\mathcal {C}$ into five subsets $\mathcal {C}^{(k)}$, $k\in \{1, \dots , 5\}$. For each $k\in \{1, \dots , 5\}$, let $\mathcal {C}_\text{train}:=\mathcal {C} \setminus \mathcal {C}^{(k)}$ and $\mathcal {C}_\text{test}:=\mathcal {C}^{(k)}$. We then construct the prediction function $\eta ^{(k)}: {\mathbb {R}}^{K}\rightarrow {\mathbb {R}}$ using $\mathcal {C}_\text{train}$ and calculate the score $\text{R}^2(\eta ^{(k)},\mathcal {C}_\text{test})$. This process is repeated for *p* times, and evaluate the model based on the median of 5*p* $\text{R}^2(\eta ^{(k)},\mathcal {C}_\text{test})$ scores.
**2.**: LOOV: For every $i\in \{1, \ldots , |\mathcal {C}|\}$, let $\mathcal {C}_\text{train}:=\mathcal {C} \setminus \{{\mathbb {C}}_i\}$, $\mathcal {C}_\text{test}:=\{{\mathbb {C}}_i\}$. Then, construct the prediction function $\eta ^{(i)}: {\mathbb {R}}^K\rightarrow {\mathbb {R}}$ based on $\mathcal {C}_\text{train}$, and calculate $\text{R}^2$ score as $\displaystyle 1- \frac{ \sum _{{\mathbb {C}}_i\in \mathcal {C}}(a_i - \eta ^i(x_i))^2 }{\sum _{ {\mathbb {C}}_i\in \mathcal {C} } (a_i-\widetilde{a})^2}.$ .
